# First presentation of a frameshift mutation in the SETD2 gene of a juvenile psammomatoid ossifying fibroma (JPOF) associated with an aneurysmal bone cyst

**DOI:** 10.1186/s13000-021-01160-w

**Published:** 2021-10-17

**Authors:** A. Toferer, A. Truschnegg, K. Kashofer, C. Beham-Schmid, A. Beham

**Affiliations:** 1 Division of Oral and Maxillofacial Surgery, Medical University of Graz, Auenbruggerplatz 5, 8036 Graz, Austria; 2 Division of Dental Medicine and Oral Health, Medical University of Graz, Graz, Austria; 3 Diagnostic and Research Center for Molecular BioMedicine, Diagnostic and Research Institute of Pathology, Medical University of Graz, Graz, Austria; 4 Diagnostic and Research Center for Molecular BioMedicine, Diagnostic and Research Institute of Pathology, Medical University of Graz, Graz, Austria; 5 Medical University of Graz, Neue Stiftingtalstraße 6, 8036 Graz, Austria

**Keywords:** Mandible, Frameshift mutation, Next-generation sequencing, SETD2 gene, Juvenile psammomatoid ossifying fibroma, JPOF, Aneurysmal bone cyst

## Abstract

**Background:**

The rarity of juvenile psammomatoid ossifying fibroma (JPOF) and lack of cytogenetic studies prompted us to report a novel SETD2 gene mutation in a benign odontogenic tumour.

**Case presentation:**

A 21-year-old man presented with a hard, expanded mandibular cortex. Computed tomography revealed multilocular radiopacity in the mandible; this was reconstructed via segmental mandibulectomy using a vascularised iliac crest flap. Based on the clinical and histological findings, we diagnosed JPOF associated with an aneurysmal bone cyst. Microscopically, the solid area was characterised by many rounded or angular ossicles in a cellular fibrous stroma. The stromal cells were spindle-like or stellate. Next-generation sequencing detected a frame shift mutation of the SETD2 gene, while the copy number was normal.

**Conclusions:**

Our findings suggest further genetic studies should be performed to assess whether this mutation is related to tumour genesis.

## Background

According to the World Health Organization (WHO) classification, there are three subtypes of ossifying fibromas of the craniofacial skeleton and jaw, differing in clinical presentation and histopathological appearance: cemento-ossifying fibroma (COF), juvenile trabecular ossifying fibroma (JTOF), and juvenile psammomatoid ossifying fibroma (JPOF) [1]. JPOFs show a slight male predominance and, in one study, the mean age of the patients was 18.9 ± 12.0 years [2]. JPOFs in the mandible are rare [2–4]. An association with aneurysmal bone cysts or cortical bone perforation with local aggressive growth has been reported in some cases [2]. Approximately one-third of JPOFs of the jaws recur; this is mainly dependent on the extent of the surgical excision [2]. The rarity of JPOF in the mandible associated with an aneurysmal bone cyst, and the lack of cytogenetic studies, prompted us to report the clinicopathological features and mutational status of such a case.

## Case presentation

**Fig. 1:**
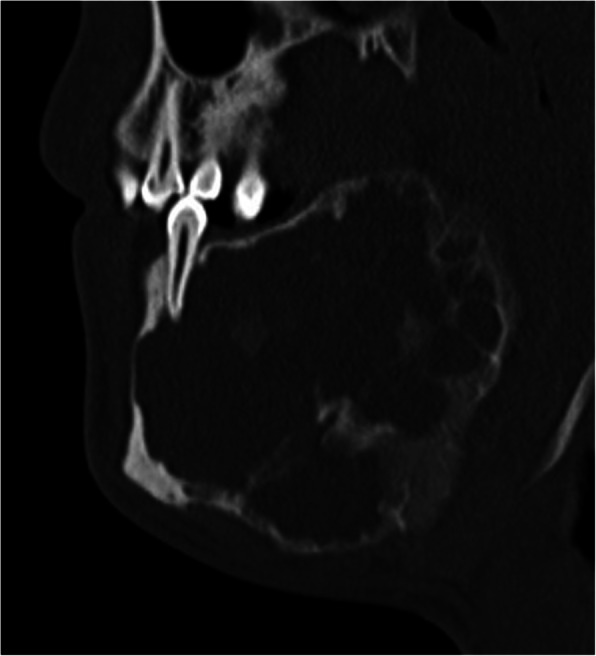
Computed tomography shows a mainly cystic lesion; the tumour proper is the solid area in the labial aspect of the lower part (arrow)

A 21-year-old man was transferred to the Department of Oral and Maxillofacial Surgery, Medical University of Graz when a slow-growing tumour in the left mandible, which had been present for 10 years and could not be diagnosed or treated adequately in his home country. On admission, he had facial asymmetry with adequate mouth opening. The intraoral examination revealed left-sided, hard, expanded buccal and lingual mandibular cortices. Computed tomography showed an 8.5-cm multilocular radiopacity in the left mandibular neck extending to the midline. Despite occupying the soft tissues, the mainly cystic, focal, solid lesion (arrow) was completely covered by thin, non-perforated, pre-existing cortical bone (Fig. 1). Laboratory work-up was normal, including endocrine parameters. Of note, the somatomedin C, calcium, and phosphate levels were within their normal ranges. The medical and family histories were non-contributory. Following a preoperative biopsy, he underwent segmental mandibulectomy for reconstruction with a vascularised iliac crest flap. The osseous flap was inserted into the defect and fixed with a plate. The postoperative healing was uneventful. After removing the plate, osseointegrated dental implants were used for dental rehabilitation. No further tumours were seen in the remaining jaw bone during a 7-year follow-up period.

### Pathology and cytogenetics

**Fig. 2:**
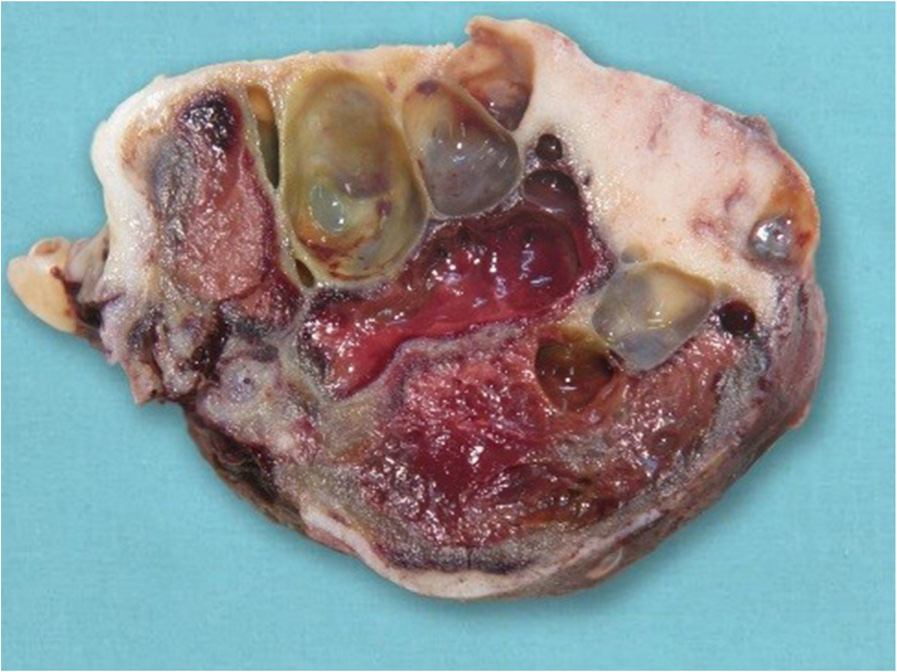
Sagittal cross-section of the tumour reveals many cystic spaces in the solid part, mirroring the computed tomography findings

**Fig. 3:**
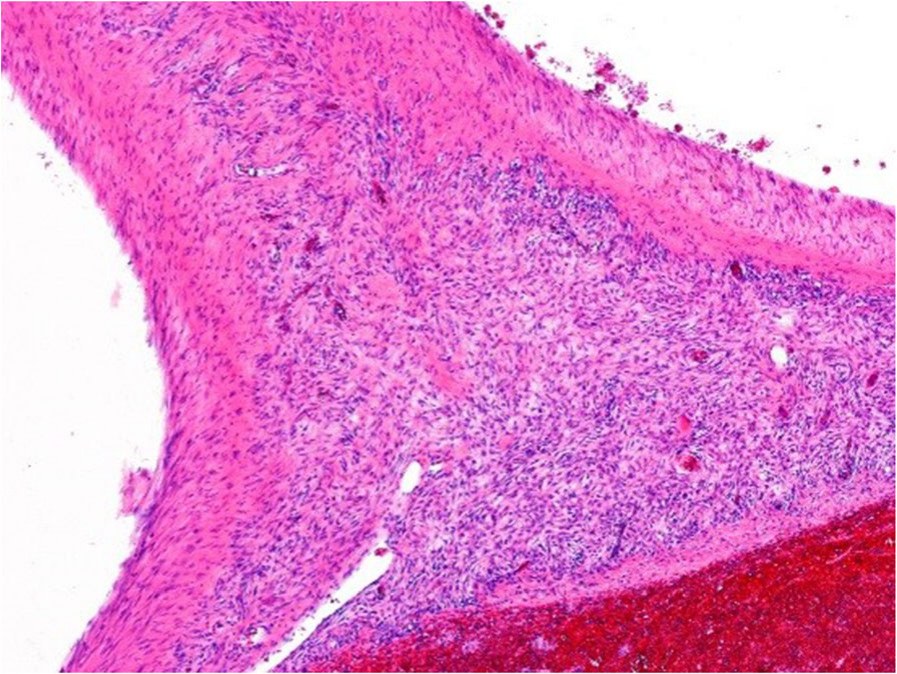
Cystic spaces (asterisks) are part of the multilocular aneurysmal bone cyst. A peripheral extension of the solid tumour is seen in the centre

**Fig. 4:**
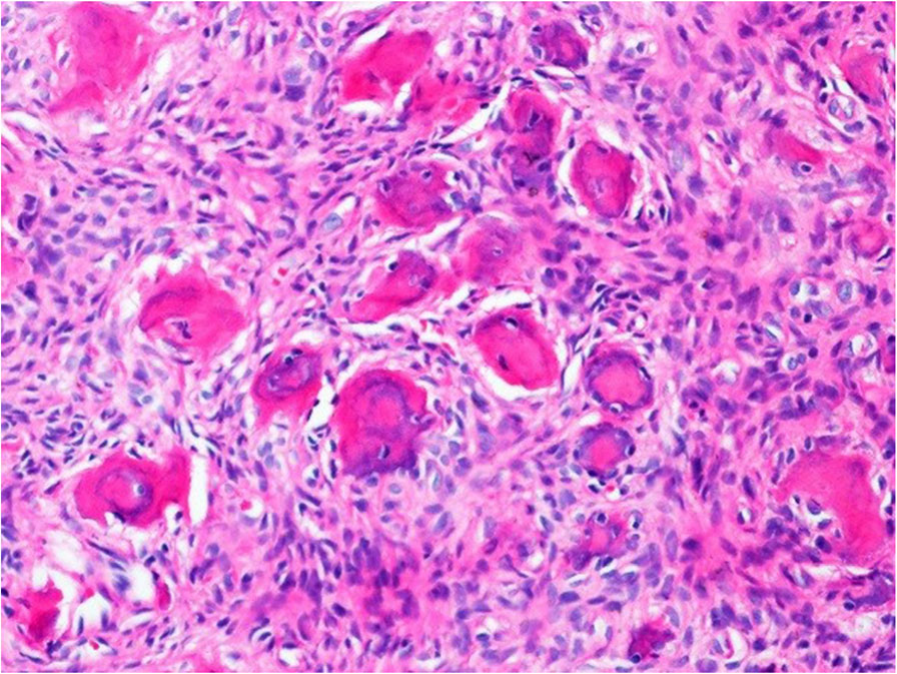
An area of the solid tumour consisting of many ossicles set in cellular stroma

**Fig. 5:**
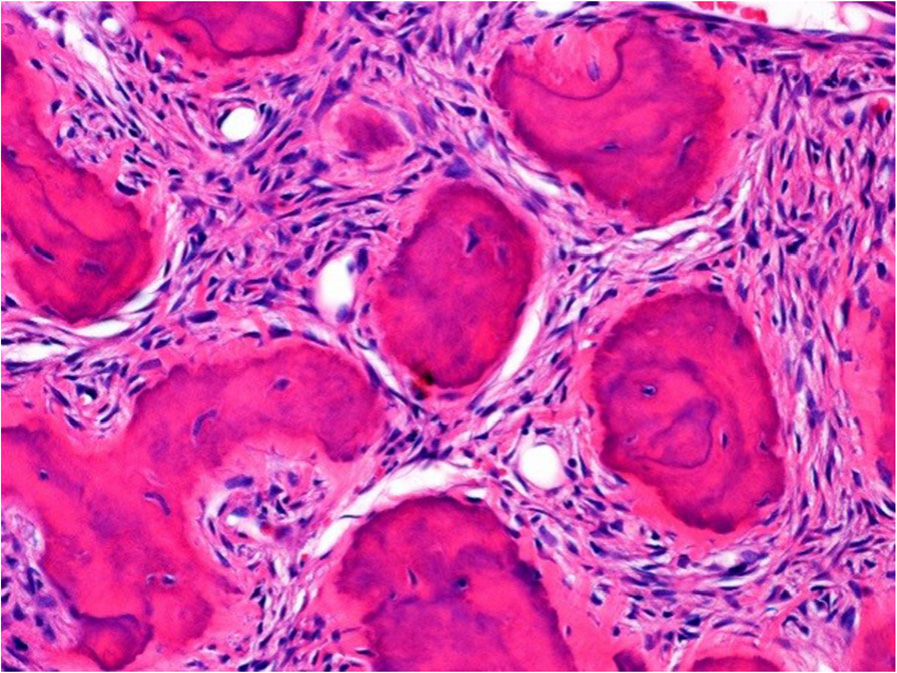
The ossicles are often rounded and show laminated calcification, reminiscent of psammoma bodies

**Fig. 6:**
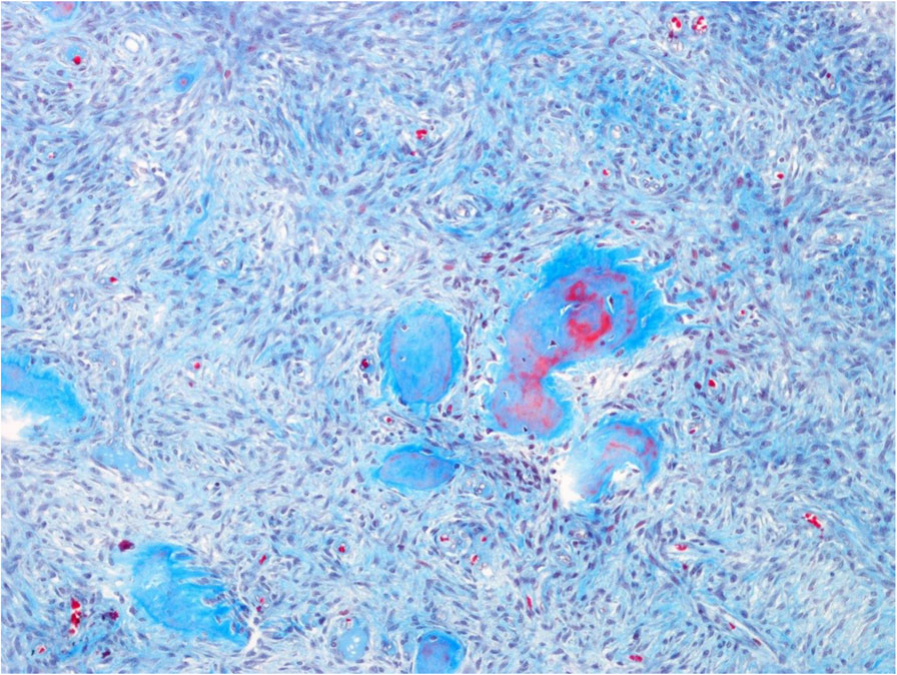
Chromotrope aniline blue staining highlights the fibrous stroma

**Fig. 7:**
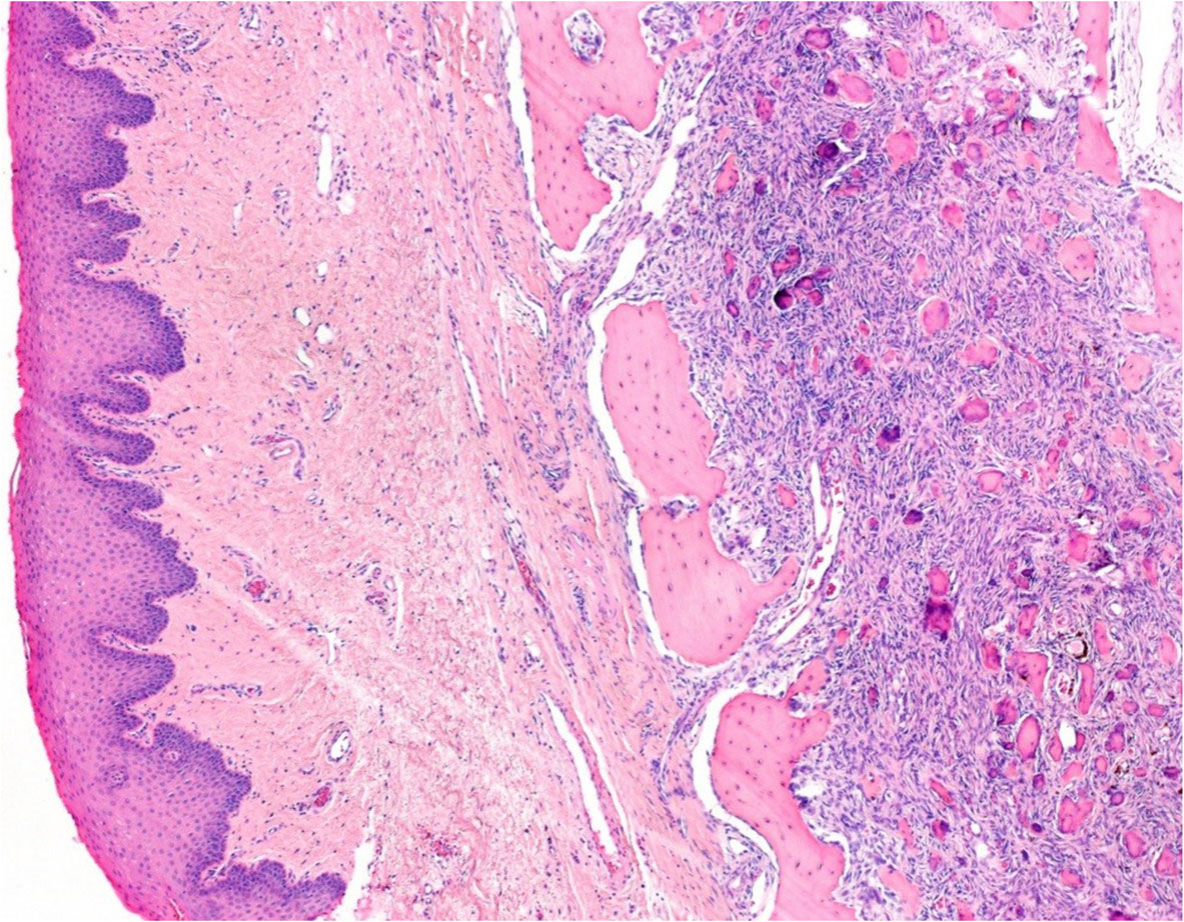
Tumour tissue infiltrating the mandibular bone, which leads to distinct osseous thinning. The bone is covered by the mucosal membrane

**Fig. 8:**
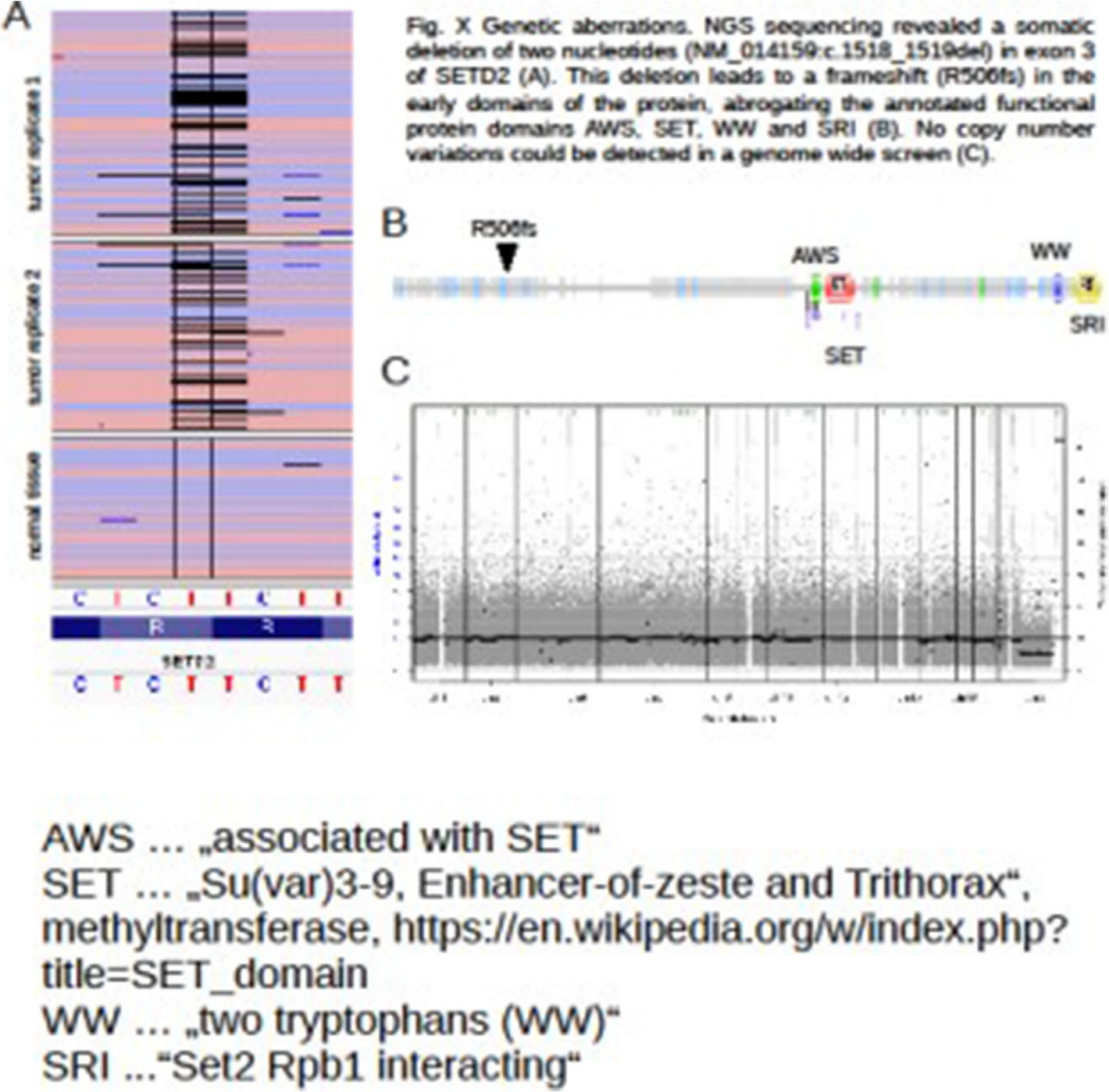
Genetic aberrations. NGS sequencing revealed a somatic deletion of two nucleotides in exon 3 of SETD2

The surgical specimen was 12 cm in its greatest longitudinal diameter and dominated by an 8-cm-diameter, nodular, bone-distending tumour. On cross-sections, the tumour consisted mainly of cystic spaces varying in size and sometimes filled with blood clots; a solid, nodular area measuring 3 cm was seen only at the lingual aspect of the lower part (Fig. 2). Microscopically, the cysts were mostly lined with granulation tissue and scar-like fibrous tissue (Fig. 3). The actual tumour, corresponding to the solid area, was characterised by many ossicles of different sizes in cellular fibrous stroma (Fig. 4). The ossicles were rounded or angular and comprised woven-bone deposits with distinct, sometimes laminated calcifications resembling psammoma bodies (Fig. 5). The stromal cells were spindle-shaped or stellate (Fig. 6). The tumour was covered peripherally by a thin shell of cortical bone, with no evidence of perforation (Fig. 7). The histopathological diagnosis of the preoperative biopsy was JPOF with an associated aneurysmal bone cyst; this was confirmed by examination of the surgical specimen, which had identical microscopic features. Paraffin-embedded tumour tissue was analysed by next-generation sequencing (NGS) using a PCR-based gene panel to detect mutations in the coding regions of 409 genes relevant to tumours, and low-density whole genome sequencing was performed to assess copy number variation. NGS showed a frame-shift mutation of the SETD2 gene, while the copy number was normal (Fig. 8).

## Discussion

Only a few JPOFs located in the mandible associated with an aneurysmal bone cyst have been reported [3–11]. The rarity of this incidental lesion is underlined by the fact that aneurysmal bone cysts comprise only 0.15–0.4% of all jaw cysts [12], with two-thirds located in the mandible [12]. To our knowledge, this is the first report to compare whole-mount sections with their imaging equivalents, highlighting the dominating cystic character of the lesion.

On searching the literature, we found no cytogenetic studies of a mandibular JPOF with an associated aneurysmal bone cyst. However, we found cases that were diagnosed retrospectively as psammomatoid ossifying fibromas on re-evaluation their histopathological appearance. One of these was located in the mandible and had an interstitial deletion on chromosome 2 between q31–32 and q35–36 [13]; three cases were in the orbit and had a chromosomal translocation t(X;2) (q26;q33) [14, 15]. Tabareau-Delalande et al. [16] presented three JPOFs of non-mandibular or unknown location, with a chromosome 12 long arm rearrangement and amplification of the MDM2 and RASR1 genes. Although not subtyping ossifying fibromas despite the introduction of JPOF as a distinct clinicopathological entity by the WHO in 2005 [1, 17], several authors have published cytogenetic studies on fibro-osseous tumours, calling them simple ossifying fibromas, COFs, or juvenile ossifying fibromas [18–28]. We speculate that some of these tumours were JPOFs. Those tumours contained mutations of the HRPT2/CDC73 gene [18, 22, 25], showed upregulated expression of Notch receptors and ligands [21], down- or upregulation of miRNA [26], or deregulation of the Wnt/ß–catenin pathway [27]. However, no GNAS [19, 20, 23, 24, 28] or HRPT2, a component of PAF1 complex, [24] gene mutations were observed. In this context, it is interesting that the first cytogenetic analysis of a gnathic ossifying fibroma was probably performed in 1992, on a cemento-ossifying fibroma of the maxilla with three chromosomal translocations [29].

Knocking out Hrpt2 in the mouse results in preimplantation lethality as well as when conditionally deleted in adult animals [30]. De Mesquita et al. found a loss of heterozygosity at the HRPT2 gene locus in ossifying fibromas, fibrous dysplasia and osteosarcomas; nonetheless, only a limited contribution to the pathogenesis was detected [31]. On the other hand, PAF1 complex is required for mammalian development, likely through regulation of H3K36me3. Knockdown of either SETD2 or RTF1 results in similar phenotypes [32]. The SETD2 gene is located on chromosome 3 p21.31 and encodes a histone methyltransferase, which is responsible for the trimethylation of lysine 36 of histone H3 to H3K36me. This is the first report of a mutation of the SETD2 gene in a JPOF for an odontogenic tumour. Furthermore, no SETD2 mutation has been detected previously in a benign tumour. Proteins listed by H3K36me readers are involved in various consecutive events, such as transcription elongation, RNA processing, and DNA repair; therefore, SETD2 is considered a tumour-suppressor gene [33, 34]. Mutations of the SETD2 gene have been found in a variety of malignant tumours, especially renal cell carcinoma [33]. SETD2 mutations have also been reported in patients with Sotos syndrome and Sotos-like syndromes [35].

## Conclusions

In our case, the SETD2 gene mutation likely played an important role in tumourigenesis. Therefore, it would be interesting to determine whether this mutation is typical of JPOF in general. To this end, further genetic studies on a JPOF series are required.

## Abbreviations

JPOF: juvenile psammomatoid ossifying fibroma
WHO: World Health Organization
CPO: cemento-ossifying fibroma
JTOF: juvenile trabecular ossifying fibroma
NGS: next-generation sequencing
